# Practices of Lyme disease diagnosis and treatment by general practitioners in Quebec, 2008–2015

**DOI:** 10.1186/s12875-017-0636-y

**Published:** 2017-05-22

**Authors:** Salima Gasmi, Nicholas H. Ogden, Patrick A. Leighton, Ariane Adam-Poupart, François Milord, L. Robbin Lindsay, Sapha Barkati, Karine Thivierge

**Affiliations:** 1 Laboratoire de santé publique du Québec, Institut national de santé publique du Québec, 20045, chemin Sainte-Marie, Sainte-Anne-de-Bellevue, H9X 3R5 Canada; 2 Policy Integration and Zoonoses Division, Centre for Food-borne, Environmental & Zoonotic Infectious Diseases, Public Health Agency of Canada, 3200 Sicotte, Saint-Hyacinthe, J2S 7C6 Canada; 3 Public Health Risk Sciences Division, National Microbiology Laboratory, Public Health Agency of Canada, 3200 Sicotte, Saint-Hyacinthe, J2S 7C6 Canada; 4 Groupe de Recherche en Épidémiologie des Zoonoses et Santé Publique (GREZOSP), 3200 Sicotte, Saint-Hyacinthe, J2S 7C6 Canada; 5 Faculty of Veterinary Medicine, University of Montreal, 3200 Sicotte, Saint-Hyacinthe, J2S 7C6 Canada; 6 Direction des risques biologiques et de la santé au travail, Institut national de santé publique du Québec, 190, boulevard Crémazie Est, Montréal, H2P 1E2 Canada; 7 Zoonotic Diseases & Special Pathogens Division, National Microbiology Laboratory, Public Health Agency of Canada, 1015 Arlington Street, Winnipeg, R3E 3R2 Canada; 8 Department of Microbiology and Immunology, Faculty of Medicine, University of Montreal, 2900, boul. Édouard-Montpetit, Montréal, H3T 1J4 Canada

**Keywords:** *Ixodes scapularis*, Lyme disease, Treatment, Diagnosis, Prophylaxis, Canada

## Abstract

**Background:**

Lyme disease (LD), a multisystem infection caused by the spirochete *Borrelia burgdorferi sensu stricto* (*B. burgdorferi*), is the most reported vector-borne disease in North America, and by 2020, 80% of the population in central and eastern Canada could live in LD risk areas. Among the key factors for minimising the impact of LD are the accurate diagnosis and appropriate management of patients bitten by ticks. In this study, the practices of Quebec general practitioners (GPs) on LD diagnosis and management of patients bitten by infected ticks are described.

**Methods:**

Eight years (2008 to 2015) of retrospective demographic and clinical data on patients bitten by infected *Ixodes scapularis* (*I. scapularis*) ticks and on the management of suspected and confirmed LD cases by Quebec GPs were analysed.

**Results:**

Among 50 patients, all the antimicrobial treatments of LD clinical cases were appropriate according to current guidelines. However, more than half (62.8%) of erythema migrans (EM) were possibly misdiagnosed, 55.6%, (*n* = 27) of requested serologic tests were possibly unnecessary and the majority (96.5%, *n* = 57) of prophylactic antimicrobial treatments were not justified according to current guidelines.

**Conclusions:**

These observations underline the importance for public health to enhance the knowledge of GPs where LD is emerging, to minimise the impact of the disease on patients and the financial burden on the health system.

## Background

Lyme disease caused by a spirochete *B. burgdorferi* is the most commonly reported vector-borne disease in North America, and is transmitted by the blacklegged ticks, *I. scapularis* and *Ixodes pacificus* [1]. In Canada, LD is emerging due to the spread of *I. scapularis* and by 2020*,* 80% of the population in south central and southeastern Canada could live in regions where LD risk areas occur [2]. In 2004, there were an estimated 40 reported human cases of LD in Canada [1]; this number rose to 917 in 2015 [3]. The same trend can be observed in the province of Quebec with the number of reported human cases increasing from 2 in 2004 to 160 in 2015 [4]. Most of the indigenous cases occurred after exposure to the Montérégie and Estrie regions [4, 5].

Lyme disease is a multisystem infection that is manifested by progressive stages. In the early stage of the disease, which lasts from several days to several weeks, a cutaneous lesion, EM appears in approximately 70% of infected patients [6]. The EM, which represents an inflammatory response to the spirochetes as they migrate through the skin away from the tick bite site, spreads to exceed 5 cm in most cases [7]. Erythema migrans can be accompanied by flu-like symptoms such as fever, fatigue, headache, myalgia, or arthralgia [6]. In many untreated cases, *B. burgdorferi* disseminates throughout the body from the skin via the blood and causes manifestations of early disseminated LD [8], which include neurological (e.g. facial palsy and meningitis), cardiac (heart block, which may on rare occasions be fatal), musculoskeletal and dermatologic (multiple secondary EM lesions) signs and symptoms [8]. Again if untreated LD can progress to the late disseminated stage, during which arthritis is the most common manifestation [9, 10].

Currently, serological testing using the two tier algorithm is the main laboratory diagnostic technique for LD in clinical practice [11]. The two-step approach, comprising a first screening enzymatic immunoassay (EIA), followed by a confirmatory Western blot test [7, 12], was developed to optimise specificity and sensitivity. However in early stages of LD, specific antibodies have not developed and serologic tests have poor sensitivity [13]. Moreover, the sensitivity of the serological tests can be negatively affected in circumstances when the patient has been successfully treated with antibiotics. Hence, in the presence of EM and when the patient has a history of possible exposure to tick vectors, laboratory testing for LD antibodies is not recommended to support diagnosis [7, 10]. The use of serologic testing is advocated to support diagnosis of early and late disseminated infection when the clinical signs can be confused with other diseases [7].

Lyme disease diagnosis is a challenge for GPs in emerging areas. Previous studies that have described the practices and knowledge of physicians have shown that: 1) the majority of GPs lack knowledge to recognize the EM [14]; 2) they do not know that EM alone is adequate for clinical diagnosis and 3) they tend to confirm the infection with laboratory tests [15, 16], even though initiation of treatment without laboratory confirmation is usually appropriate [17, 18].

In this 8-year retrospective study, data on the management of tick-bitten patients by GPs in Quebec were used to analyze their knowledge and practices regarding: 1) the recognition of early LD clinical signs; 2) appropriateness of prophylactic treatment; 3) treatment of LD cases and 4) laboratory tests requested. Evidence-based guidelines for the management of patients with LD, published in 2006 by the Infectious Diseases Society of America (IDSA) were used as the reference for recommended approaches to LD diagnosis and treatment [10].

## Methods

### Data

Data for the study were collected by the Quebec LD passive tick surveillance system from 2008 to 2015. The collection, use, analysis, and disclosure of data described in the current article fall within the surveillance mandate. Therefore, research ethics committee approval was not required. Medical and veterinary clinics participating in this surveillance system submit their ticks to the Laboratoire de santé publique du Québec (LSPQ). The species of the tick is identified at the LSPQ and the state of engorgement (categorised as not engorged or evidence of engorgement) as well as the developmental stage (nymph, larva or adult) are recorded. Ticks identified as *I. scapularis* are sent to the National Microbiology Laboratory of the Public Health Agency of Canada for detection of *B. burgdorferi* by polymerase chain reaction [19]. For the current study, the data were those from human patients that had been bitten by ticks and submitted by medical clinics. When ticks were PCR positive for *B. burgdorferi*, a simple questionnaire was sent to the GPs by the LSPQ to collect clinical (onset of illness, and presence of EM and/or presence of manifestations of disseminated LD affecting the nervous system, cardiac system and/or musculoskeletal system) and demographic (sex and age) information as well as details on how the person was being managed with respect to treatment and diagnostic testing. The questionnaires were sent 6 to 12 weeks after the reception of the tick for identification at the LSPQ. The populations of interest in this study were: 1) the patients in Quebec bitten by a detectably *B. burgdorferi*-infected *I. scapularis* tick and 2) the GPs of those patients.

### Descriptive analysis

The practices of GPs, between 2008 and 2015, with respect to patients bitten by an infected blacklegged tick were described. A possible case of LD was defined in the present study as a patient presenting with objective manifestations of disseminated LD (neurological, cardiac or musculoskeletal clinical sign) or of early LD (EM), according to the GP [10]. Erythema migrans usually develops between 7 and 14 days after a tick bite and may persist for several weeks [10, 20]. Any diagnosis of EM reported by the GPs in the present study less than 5 days after the tick bite likely represent hypersensitivity reactions to the tick bite and not genuine EM [21]. This 5 days threshold was used to assess clinicians’ knowledge of the characteristics EM.

One option for prevention of LD after a tick bite is the use of antimicrobial prophylaxis. The knowledge of GPs regarding prophylactic antibiotic use for tick bites was assessed against the IDSA guidelines [10]. According to these guidelines, one dose of doxycycline can be administered to a patient bitten by a tick if all the following conditions are met: 1) the tick can be identified as an adult or nymphal *I. scapularis* tick (or *I. ricinus* in someone who has travelled to Europe where this tick occurs) that is estimated to have been attached for at least 36 h on the basis of the state of the tick’s engorgement; 2) prophylaxis can be initiated within 72 h of the time that the tick was removed; 3) the infected tick was acquired in an area where the prevalence of *B. burgdorferi* is likely to be at least 20% and 4) doxycycline is not contraindicated for the patient (i.e. children under 8 years of age and pregnant women) [9, 10]. During the study period, Quebec GPs were not recommended to prescribe prophylactic therapy to patients bitten by a tick acquired in the province because it was considered that the prevalence of infection in ticks in Quebec was below the 20% threshold for prophylactic treatment [10]. In contrast, patients who reported a history of travel to the USA, Europe and in areas of Canada where tick infection prevalence may be ≥ 20% could receive medical prophylaxis [20]. All descriptive analyses were performed in IBM SPSS statistical software (version 23).

## Results

### Physicians’ knowledge of LD clinical signs

**Fig. 1:**
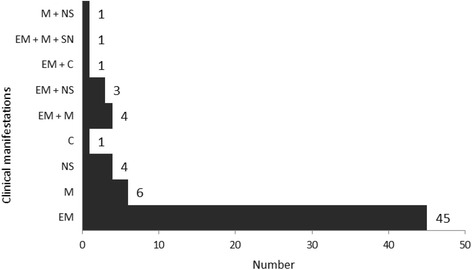
Frequency of clinical manifestations reported by GPs for patients bitten by infected *I. scapularis* (EM: erythema migrans; M: musculoskeletal system; NS: nervous system; C: cardiovascular system)

There were 495 people bitten by *I. scapularis* ticks that were infected with *B. burgdorferi*. The data on clinical information collected by a questionnaire administered to the GPs were available for 254 patients (51.3%). Of these, 66 (26.0%) were diagnosed as having LD by their physician and these included 54 who were diagnosed with EM (Fig. 1). Twenty-one persons were reported to have manifestations of disseminated clinical signs of LD by their GPs with seven cases of neurological involvement, ten cases of musculoskeletal involvement, two case of cardiac involvement and two with both neurological and musculoskeletal manifestations (Fig. 1).

**Fig. 2:**
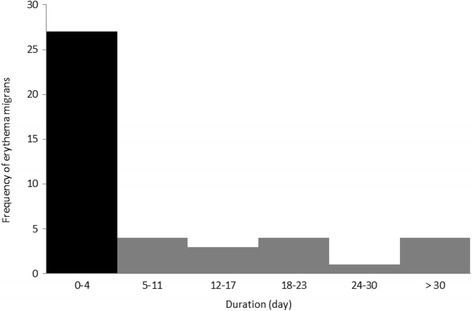
Frequency of EM diagnoses in relation to time elapsed between diagnosis of EM and the date of tick removal. The *black bar* shows the frequency of reported EM cases that were considered to be misdiagnosed (diagnosed < 5 days from the date of tick bite)

However, 27/43 (63%) of these 54 persons for which the information was available had skin lesions diagnosed as EM even though they occurred less than 5 days from the date of tick bite and as a result were likely hypersensitivity reactions and not EM (Fig. 2) [10]. Indeed, 44% of EM lesions were diagnosed at the time of tick-removal while 9% of EM were diagnosed beyond 1 month post infection (Fig. 2).

Once reported disseminated LD cases for which we do not have laboratory confirmation or who have been treated are removed from the analysis, and only patients that reported “true” EM (diagnosed ≥ 5 days from the date of tick bite) are considered the proportion of people bitten by an infected tick that subsequently developed manifestations consistent with LD was 6.7% (16/239, 95% CI = 3.9–10.6).

### Prophylactic treatment

Amongst asymptomatic patients bitten by infected *I. scapularis* and for which treatment information was available (147/188), 38.8% received antimicrobial prophylaxis. The proportions of these treatments for which the patients met each of the four IDSA criteria for prophylactic treatments are shown in Table 1.

**Table 1:** Frequency and proportion of criteria met simultaneously to justify prophylaxis as recommended by IDSA guidelines

Sum of criteria	Number of patients	Relative frequency (%)	Cumulative frequency (%)
0	2	3.5	3.5
1	19	33.3	36.8
2	23	40.4	77.2
3	11	19.3	96.5
4	2	3.5	100.0
Total	57	100	

Among patients receiving prophylactic treatments, only 3.5% (2/57) met the four criteria. Moreover, among the 55 (96.5%) patients for whom the four criteria were not met simultaneously, the analysis of each criterion showed that: 1) 49.1% of patients were treated beyond 72 h after the tick was removed; 2) 83.6% of ticks had likely not fed for ≥ 36 h (i.e. the ticks were non-engorged); 3) 81.8% of the ticks were acquired in areas where the prevalence of *B. burgdorferi* in blacklegged ticks was lower than 20% and 4) 7.3% of patients were younger than 8 years of age. These proportions assume that when prophylactic treatments were given they were the recommended one dose of doxycycline because the antibiotic used was not recorded for 41/57 prophylactic treatments. For the 16 patients receiving prophylaxis for which the antibiotic treatment was recorded, there were 14 adults who received doxycycline and 2 children < 8 years who were treated with amoxicillin. Four of the treatments were administered as a course of several days of antibiotics.

### LD cases treatments

Recommended treatments for early stage of LD, with EM, are doxycycline (100 mg twice per day for 10 to 21 days for those for whom doxycycline is not contraindicated), amoxicillin (500 mg 3 times per day for 14 to 21 days) or cefuroxime axetil (500 mg twice per day for 14 to 21 days) [10]. All patients diagnosed with at least one clinical manifestation of LD for whom the type of antibiotic was recorded (*n* = 14) received one of the three recommended antibiotic treatments.

### Serologic tests requests

**Fig. 3:**
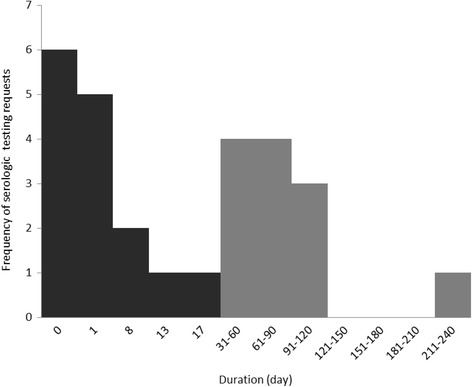
Frequency of serologic testing requests in relation to time elapsed between the date of serologic testing requests and the date of tick removal

When EM occurs in a patient with a history of tick exposure, this information is sufficient for diagnosis, and serologic testing for *B. burgdorferi* antibodies is not recommended [7]. The present study showed that among 66 patients diagnosed with LD and for whom data on dates of a first serological tests requests are available (*n* = 27), more than half (55.6%) of serological testing for LD was requested within 18 days of the tick bite. Of these, 40.7% serological testing were requested the same day or the day after the tick was removed (Fig. 3).

For 50 (26.6%) of 188 patients bitten by infected blacklegged tick who did not show LD clinical signs a serologic test was requested. A second serologic test was requested for 5.3% of these patients.

## Discussion

Of those patients bitten by detectably *B. burgdorferi*-infected *I. scapularis* who were then diagnosed as having LD, almost one half were diagnosed as having EM less than 5 days after the tick was detected (and most of them were diagnosed at the moment of tick-removal) and we considered it likely that the skin lesions seen by the doctor were in fact reactions to the tick bite [10, 21]. It was assumed that reported EM cases fulfilled other criteria for diagnosis of EM such as having a diameter of > 5 cm [10]. A recent cross-sectional study has shown a moderate lack of knowledge of Lyme disease by family physicians in southern Quebec [16], suggesting that increased physician awareness may reduce the likelihood of such misdiagnoses in the future.

In our study, more than half of the GPs are practicing in the Montérégie and Estrie health regions, where public health authorities have provided GPs with information on LD since 2010. The over-diagnosis evident in this study is probably due to the combination of lack of knowledge and high risk perception among these GPs. The possible negative consequences of over-diagnosis of EM are stress and adverse effects of antibiotics for the patient, an overuse of resources [22], generating costs to the health system [23], and probably a contribution to antibiotic resistance [24, 25]. Evidence of lack of knowledge on Lyme disease is present despite efforts to provide information to practitioners suggests that some more novel methods of practitioner education may be needed.

However, in the other health regions of Quebec, where GPs have not yet received information to raise their awareness of LD, and where the risk for the population is lower than in Montérégie and Estrie health regions, EM may go unnoticed [26]. An accurate diagnosis of early infection is sufficient to administer an antimicrobial treatment at a stage when the spread of the pathogen in the blood to other organs can, in most cases, be avoided [10]. Not recognizing early LD clinical signs or delay in diagnosis could complicate the diagnosis and consequently require more intensive and longer-term antibiotic courses for the patient [27].

The present study showed that few patients were diagnosed, by Quebec GPs, with EM beyond a month after exposure, but these could have been multiple EMs, which may appear up to 70 days post infection as part of the spectrum of manifestations of early disseminated LD [21]. This result may indicate that Quebec GPs do recognize multiple EM as a manifestation of LD but further studies are needed to confirm this.

Previous estimates of risk of occurrence of LD after an infected *I. scapularis* tick-bite range from 3.7% to 6% [28, 29]. However, this study has shown that approximately one quarter of patients bitten by infected *I. scapularis* were diagnosed as having LD cases by Quebec GPs. This difference could be explained by the over diagnosis of EM. Moreover, reports of confirmatory laboratory testing for the reported cases of disseminated LD in our study were scant and it is also possible that the number of disseminated LD cases was over-estimated due to neurological, cardiac or musculoskeletal manifestations caused by clinical conditions other than LD. When non-genuine EM and non-laboratory confirmed cases of disseminated LD were removed from the analysis, the proportion of bitten patients with infected *I. scapularis* who developed LD was similar to that estimated by Huegli et al. [29].

The results of this study showed that Quebec GPs are currently challenged in their practices regarding patients bitten by a tick in this area of LD emergence. The majority of prophylactic treatments of LD were not justified suggesting a lack of knowledge of recommended best practices [10]. The unjustified prescription of antibiotic prophylaxis by Quebec GPs in the present study could be influenced by the intention to prevent LD or other infections transmitted by a tick bite or to yield to patients’ requests [30, 31]. Furthermore, some prophylactic treatments were not aligned with the guidelines regarding the type of antibiotic prescribed and when the appropriate antibiotic was prescribed some therapeutic protocol did not follow the recommendations regarding the duration of treatment [9, 10].

In contrast, this study showed that all the treatments prescribed for cases suffering LD were appropriate, regarding the choice of antimicrobial, as reported in other studies [17, 18].

The diagnostic accuracy of serologic assays is dependent on multiple factors including timing of specimen collection with respect to disease state. For LD cases, as diagnosed by Quebec GPs, more than half of serological tests were requested less than 18 days from the date of tick removal. Serological evaluation for antibodies to *B. burgdorferi* following removal of an attached tick or soon after EM is noticed is not helpful diagnostically – results are often negative and therefore of limited clinical utility. [7, 12]. This tendency for GPs to attempt to confirm the diagnosis of EM by a laboratory test has been reported among clinicians in British Columbia and in the state of New Hampshire, USA [15, 17]. The LD serologic testing is generally recommended in late LD when disseminated clinical signs could be confused with other diseases [12].

For approximately one quarter of patients bitten by infected *I. scapularis* who never showed clinical signs of LD during 3 months post exposure, one or more serological tests were requested. Serological tests should be used to help diagnosis in cases of disseminated LD, but in the absence of objective clinical signs, serological testing requests would be unjustified and are discouraged [9, 10]. These findings suggest that Quebec GPs lack knowledge on appropriate practices regarding serologic testing for asymptomatic patient bitten by infected *I. scapularis* as reported in Ferrouillet et al. [16].

There are, of course, limitations to this study, the foremost of which is the representativeness of the sample. Data were collected by a simple questionnaire completed by GPs who received a positive *B. burgdorferi* result for the analysis of a tick collected from the patient, and it could be argued that this test result influenced the practices of the GP regarding serological testing requests and treatment. In a recent study 69% of GPs in the study region considered that sending a tick taken from patient to the laboratory for identification and detection of *B. burgdorferi* was useful for the diagnosis of LD [16]. However, the tick identification and testing described here (and previously) is a surveillance tool and not a diagnostic procedure so there is 6–12 week delay between tick collection and return of test results. So the tick PCR test result would not have impacted practices of testing and treatments (including tick bite prophylactic treatments) prior to this. We also do not know if the practices of the GPs who completed the questionnaire are representative of the practices of all GPs in the province, and it could be argued that the GPs who participate in the surveillance, sent ticks for analysis and completed the questionnaire may be those with most awareness of Lyme disease. The time between tick removal and follow-up questionnaire varied from patient to patient which may have influenced the information available at the time of completion of the questionnaire. Moreover, the ≥5 days threshold for classifying an EM as a genuine EM was based on the date of tick removal without taking into account the time of tick attachment, however only a small proportion of ticks (16%) showed the signs of engorgement that would suggest they had fed for several days so we do not think we are overestimating the number of likely false EMs.

## Conclusions

The present study showed that Quebec’s GPs are not familiar with early LD clinical signs and have tendency to confirm the clinical diagnosis by serologic testing when this is not recommended. Moreover, some GPs request serological tests for bitten patients even if no clinical sign was manifested. The majority of prophylactic treatments were not justified mainly because the removed tick hadn’t been attached to the patient long enough to transmit the causative agent of LD and also because most of the ticks were acquired in areas where the prevalence of *B. burgdorferi* was lower than 20%. However, the study showed appropriate practices regarding the type of antibiotic used for treatment of LD cases and diagnosis of multiple cutaneous manifestations.

Lyme disease is a public health concern that requires a multidisciplinary approach to reduce an increase in human incidence and to limit its impact in infected patients. It should be noted that for GPs to adhere to current guidelines requires that knowledge of the ecology of transmission cycles (specifically infection prevalence in ticks) is communicated to GPs and that the latter are trained to identify the species of the tick and its engorgement state.

Our study suggests that public health authorities need to target front-line health professionals with education on LD and on evidence-based guidelines for the management of exposed patients to avoid overuse of public health resources and for the benefit of the health of the population.

## Abbreviations

*B. burgdorferi*: *Borrelia burgdorferi*
EIA: Enzymatic immunoassay
EM: Erythema migrans
GP: General practitioner
*I. scapularis*: *Ixodes scapularis*
IDSA: Infectious Diseases Society of America
LD: Lyme disease
LSPQ: Laboratoire de santé publique du Québec
